# Targeted Drug Therapy for Senescent Cells Alleviates Unilateral Ureteral Obstruction-Induced Renal Injury in Rats

**DOI:** 10.3390/pharmaceutics16060695

**Published:** 2024-05-23

**Authors:** Ting Li, Kexin Yang, Yinghao Tong, Shangze Guo, Wei Gao, Xiangyu Zou

**Affiliations:** School of Basic Medical Sciences, Shandong Second Medical University, Weifang 261053, China; 20220115@stu.wfmc.edu.cn (T.L.); 20220118@wfmc-edu.cn (K.Y.); 20230109@stu.wfmc.edu.cn (Y.T.); 20230112@stu.wfmc.edu.cn (S.G.)

**Keywords:** cellular senescence, UUO, renal injury, ABT263, targeted drug therapy, drug delivery

## Abstract

Hydronephrosis resulting from unilateral ureteral obstruction (UUO) is a common cause of renal injury, often progressing to late-stage renal fibrosis or even potential renal failure. Renal injury and repair processes are accompanied by changes in cellular senescence phenotypes. However, the mechanism is poorly understood. The purpose of this study is to clarify the changes in senescence phenotype at different time points in renal disease caused by UUO and to further investigate whether eliminating senescent cells using the anti-senescence drug ABT263 could attenuate UUO-induced renal disease. Specifically, renal tissues were collected from established UUO rat models on days 1, 2, 7, and 14. The extent of renal tissue injury and fibrosis in rats was assessed using histological examination, serum creatinine, and blood urea nitrogen levels. The apoptotic and proliferative capacities of renal tissues and phenotypic changes in cellular senescence were evaluated. After the intervention of the anti-senescence drug ABT263, the cellular senescence as well as tissue damage changes were re-assessed. We found that before the drug intervention, the UUO rats showed significantly declined renal function, accompanied by renal tubular injury, increased inflammatory response, and oxidative stress, alongside aggravated cellular senescence. Meanwhile, after the treatment with ABT263, the rats had a significantly lower number of senescent cells, attenuated renal tubular injury and apoptosis, enhanced proliferation, reduced oxidative stress and inflammation, improved renal function, and markedly inhibited fibrosis. This suggests that the use of the anti-senescence drug ABT263 to eliminate senescent cells can effectively attenuate UUO-induced renal injury. This highlights the critical role of cellular senescence in the transformation of acute injury into chronic fibrosis.

## 1. Introduction

Acute kidney disease (AKI) is a common clinical renal disease characterized by rapidly reduced kidney function within a short period, manifested by azotemia, disturbances in water–electrolyte and acid–base balance which can affect the normal functioning of other organs or systems in the body such as the lungs, heart, brain, and intestines [1,2,3]. AKI occurs due to various causes, including decreased renal blood flow, tubular injury, and glomerular disease [4]. Hydronephrosis caused by unilateral ureteral obstruction (UUO) is a common cause of AKI [5]. Left unaddressed, this can progress to late-stage kidney fibrosis or even renal failure [6]. Studies have shown that when the kidney is damaged beyond its compensatory capacity, its normal repair process will be interfered with, leading to a variety of pathological changes in the kidneys, such as renal tubular atrophy and inflammatory cell infiltration [7,8,9]. Therefore, timely treatment of and intervention in renal disease is essential to avoid disease progression and serious consequences.

In clinical work, there are appropriate treatment options and strategies for AKI and chronic kidney disease (CKD). However, it is of concern that the transition from acute injury to chronic fibrosis is often overlooked. This transition has important implications for the progression of kidney disease, and it involves multiple complex pathophysiological mechanisms, including apoptosis, inflammatory responses, oxidative stress, and fibrotic processes. Understanding the interactions and regulatory mechanisms of these processes is essential for the development of appropriate therapeutic regimens and strategies, whereas there is a lack of validated biomarkers to predict the AKI-CKD transition, which can guide the participation of patients with early AKI in clinical trials to prevent this transition [10]. It has been well-documented that cellular senescence plays a role in AKI and CKD [11]. Therefore, it is reasonable to speculate that cellular senescence is a key factor in the transition from acute injury to chronic fibrosis.

Cellular senescence is an important component of organismal aging, playing a key role in the development of renal diseases [12]. The main features of cellular senescence include cell cycle arrest, which impairs the ability of damaged cells to produce offspring normally [13]. Senescent cells have been found extensively in aged and diseased tissues of several species, with higher expression levels than in normal animal tissues [14]. Transgenic technology has demonstrated that senescent cells can drive disease development and accelerate disease progression [15]. In animal AKI models, many senescent renal tubular cells expressing p16 and p21 can be observed early on, and these cells are thought to play a role in acute tissue repair processes [16]. However, if cellular senescence is aberrantly activated, it may lead to persistent inflammation and fibrosis formation [17]. This suggests that the decreased regenerative capacity after acute injury is associated with cellular senescence mechanisms. Cellular senescence may lead to an accumulation of intracellular deoxyribonucleic acid (DNA) damage, further triggering processes such as inflammatory response, oxidative stress, and extracellular matrix deposition, and accelerating the process of fibrosis [18]. Therefore, acute injury-induced cellular senescence may be an important pathophysiological mechanism leading to the transition from early acute injury to chronic fibrosis.

Anti-senescence agents have been found to selectively eliminate senescent cells in animal models, alleviating chronic diseases including pulmonary fibrosis, vascular calcification, Alzheimer’s disease, hepatic steatosis, and obesity [19]. Among them, ABT263 (B-cell lymphoma-2 specific inhibitor, Bcl2 specific inhibitor) is a potent anti-senescence drug that can eliminate senescent cells in a selective, efficient, and rapid manner [20]. Further studies are needed to determine whether the anti-senescence drug ABT263 can alleviate the progression from early acute injury to chronic fibrosis induced by UUO.

In this study, we examined the changes in cellular senescence phenotypes during the early stages of UUO-induced acute injury progressing to chronic renal fibrosis. After using the anti-senescence drug ABT263 to modify the senescence-associated phenotypes, we assessed the levels of inflammation, oxidative stress, fibrosis, and other indicators in the kidneys after the drug intervention. The aim was to investigate whether the removal of senescent cells using the ABT263 could play a role in the repair of renal injury. The results showed that cellular senescence did occur during the progression of UUO-induced acute injury into chronic renal fibrosis, and the ABT263 intervention was effective in eliminating senescent cells, along with reduced levels of inflammation and oxidative stress, which were beneficial to the repair of renal injury.

## 2. Materials and Methods

### 2.1. Animals

The experimental animals used in this study were male Sprague Dawley (SD) rats, 6 weeks old, and were purchased from Jinan Pengyue Laboratory Animal Breeding Company (Jinan, China). The rats were positioned in a conventional house with conventional bedding, 12:12 h light/dark cycles, and controlled ambient temperature, and had free access to food and water. All our animal experiments were approved by the Ethics Committee for Laboratory Animals of Shandong Second Medical University, and the procedures for surgery and necropsy were performed in strict accordance with the international guidelines for the use of laboratory animals.

### 2.2. UUO and ABT263 Treatment

SD rats were randomly divided into three groups, namely the Sham group, UUO group (1 d, 2 d, 7 d, 14 d), and ABT263 group. The rats were fasted overnight before surgery and water intake was free. To ensure minimal discomfort in rats, they were anesthetized using a 0.7% chloral hydrate solution before undergoing surgery. Post-anesthesia, the rats were kept warm on a mat prior to the surgical procedure. In the sham surgery group, we opened the abdomen of the rats to expose the left kidney, without ligation of the ureter. In contrast, the UUO group underwent the opening of the abdomen to expose the left kidney and subsequently the ligating of the left ureter with silk.

ABT263 (MedChemExpress, MCE, Princeton, NJ, USA) was dissolved in 10% ethanol, 30% polyethylene glycol 400 (PEG400), and 60% Phosal 50 PG. Intermittent administration started on postoperative day 2 at a dose of 50 mg/kg by gavage.

### 2.3. Senescence-Associated β-Galactosidase Staining

A senescent cell β-galactosidase staining kit (Servicebio) was used in the analysis. Frozen sections were reheated at room temperature. β-galactosidase staining fixative was added dropwise to completely cover the tissue and it was washed with phosphate-buffered solution (PBS). The wet box was incubated until the senescent cells of the tissue appeared in color. After removing the staining solution, the tissue was rinsed sequentially with PBS and purified water. Anhydrous ethanol was dehydrated, and the sections were sealed with neutral resin.

### 2.4. qPCR

RNA was extracted from kidneys using a Total RNA Extraction Kit (Solarbio, Beijing, China) according to the manufacturer’s instructions and was then reverse-transcribed to cDNA (TOYOBO, Osaka, Japan). qPCR was carried out on an Applied Biosystems QuantStudio 1 Plus (Waltham, MA, USA) using a standard protocol. Primer information is provided in Table 1.

### 2.5. Western Blot

Kidney tissues were lysed with RIPA lysis buffer (Servicebio, Wuhan, China) and centrifuged, and then the protein samples were quantified using the BCA Protein Assay Kit (Thermo Scientific, Waltham, MA, USA). Proteins were separated on SDS gels and transferred to PVDF membranes. The membranes were then incubated with primary antibody, followed by incubation with anti-rabbit secondary antibodies (1:8000, Proteintech, Wuhan, China). Membranes were treated with an ECL blotting substrate and then were imaged and quantified using ImageJ 1.54d software. The following proteins were detected by Western blot: Bcl2 (1:2000, ABclonal, Wuhan, China), Bcl2-associated X (Bax) (1:2000, Proteintech), Cleaved-caspase3 (C-Caspase3) (1:1000, Proteintech), P16 (1:2000, Abcam, Cambridge, UK), P21 (1:2000, Abcam), and Klotho (1:1000, Affinity, Liyang, China).

### 2.6. Renal Function Assay

After overnight incubation at 4 °C, whole blood was centrifuged and the supernatant was taken for testing. Serum creatinine (Scr) and blood urea nitrogen (BUN) were measured according to the specific instructions of the kit.

### 2.7. Histopathology

Kidneys were fixed in 4% paraformaldehyde and paraffin-embedded sections were stained with Hematoxylin and Eosin staining (HE). Sections were observed under the microscope to find the tubular injury, with particular attention paid to dilatation, necrosis, brush border shedding, and the formation of tubes in the lumen. Based on the scope of tubular injury, the semi-quantitative scores of tubular injury were as follows: 0, no injury; 1 point, <25%; 2 points, 25~50%; 3 points, 50~75%; 4 points, >75%.

### 2.8. Immunohistochemistry

Kidney sections were incubated with primary antibodies against α-SMA (1:100, Servicebio), Ki67 (1:200, Servicebio) and CD68 (1:50, Servicebio) overnight at 4 °C, followed by incubation with the corresponding secondary antibodies for 1 h at room temperature.

### 2.9. Immunofluorescence

Kidney sections were positioned in 4% paraformaldehyde and then permeabilized with 0.2% Triton X-100 in PBS. Serum was blocked, and sections were immunostained with primary antibody against Lamin B1 (Abcam). To visualize the primary antibody, slides were stained with FITC-coupled secondary antibody.

### 2.10. Measurement of Superoxide Dismutase (SOD) and Myeloperoxidase (MPO)

Rat kidney tissues were homogenized and ground by adding an appropriate amount of saline. The mixture was then centrifuged, and the supernatant was taken. The expression of SOD and MPO was detected by adding the corresponding reagents (Servicebio) according to the instructions.

### 2.11. Statistical Analysis

Data were expressed as means ± SEM for at least three independent experiments. Group comparisons were then conducted using one-way ANOVA, with *p* values < 0.05 considering statistically significant. *** *p* < 0.001, ** *p* < 0.01, * *p* < 0.05.

## 3. Results

### 3.1. Structural and Functional Damage to the Kidney Tissue

**Figure 1:**
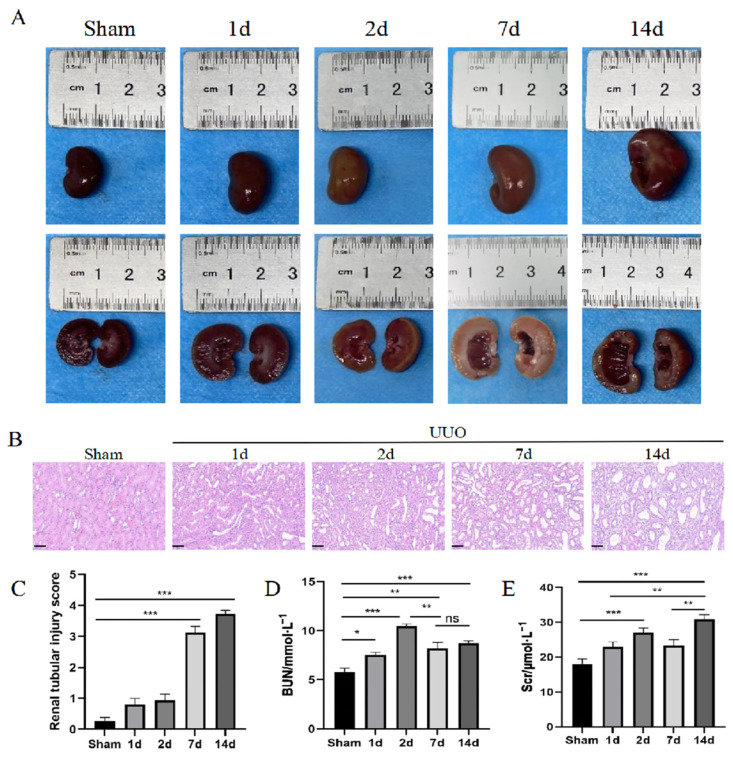
Kidney tissue structure and functional damage. (**A**) Renal appearance at different time points after UUO. (**B**) HE staining. Scale bar = 50 μm. (**C**) Renal tubular injury score. (**D**,**E**) Quantification of BUN and Scr. Data were expressed as means ± SEM for at least three independent experiments. *p* values were calculated using one-way ANOVA relative to the sham group. *** *p* < 0.001, ** *p* < 0.01, * *p* < 0.05 indicate the statistical significance compared to the sham group. ns indicates no statistical significance.

At the time of postoperative sampling, the appearance of the kidneys had changed significantly. Upon visual observation, the size and morphology of the kidneys in the sham group were normal, while in the UUO group, the kidneys on the obstructed side were enlarged, cystic, and contained brown turbid urine. Additionally, the renal parenchyma in the UUO group was thinned and accompanied by the appearance of irregularities or areas of sclerosis on the surface (Figure 1A). There were no obvious changes in the UUO group at the early stage of the injury. However, with the prolongation of the obstruction, the kidneys underwent significant changes by day 7. The tissue examination revealed diffuse renal interstitial inflammatory cell infiltration and interstitial edema, accompanied by varying degrees of renal tubular epithelial cell swelling, vacuolar degeneration, and the dilatation of the lumen of some tubules, which might necrolyze and detach into the tubular lumens (Figure 1B,C). Meanwhile, in the sham group, the renal interstitium occasionally showed a vacuolar degeneration of the tubular epithelial cells. Compared to the sham group, the BUN and Scr levels were significantly higher in the UUO group. While these levels tended to decrease on day 7, they remained higher than in the sham group, and then increased significantly (Figure 1D,E). This also reveals that the kidney may undergo a transition from acute to chronic injury after UUO.

### 3.2. UUO Causes Cellular Senescence Accompanied by Senescent Phenotypic Changes

**Figure 2:**
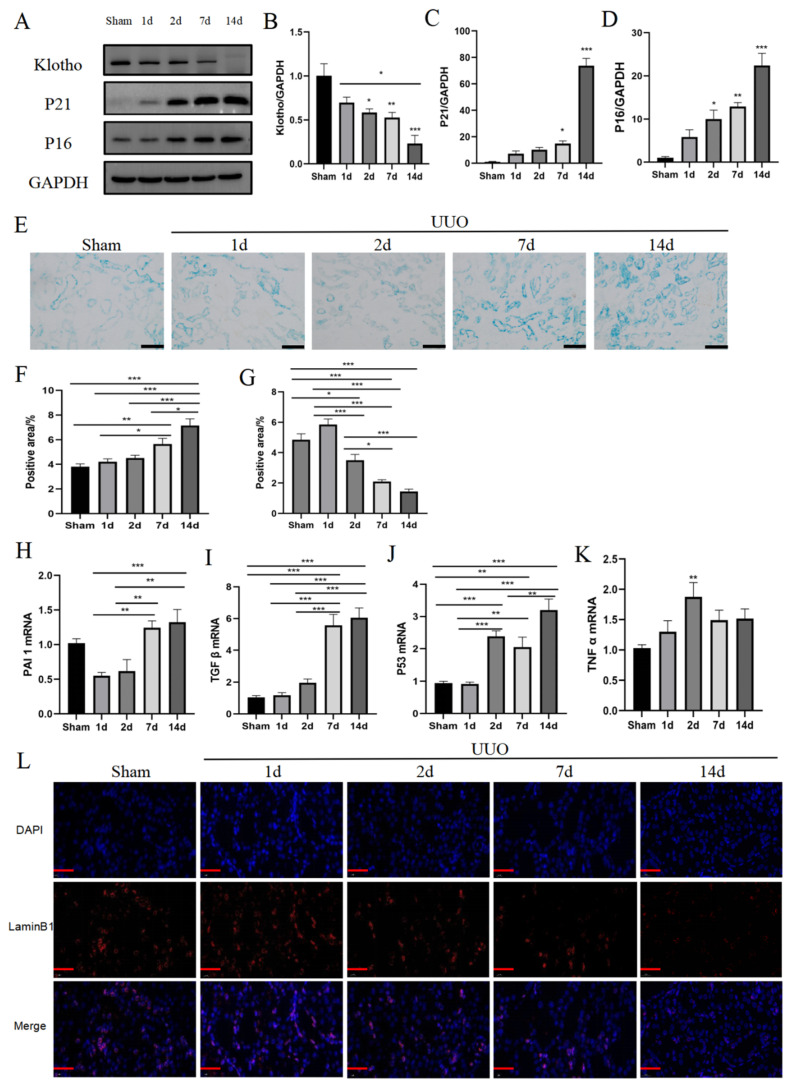
UUO causes cellular senescence, accompanied by senescent phenotypic changes. (**A**) Representative immunoblot images of Klotho, P21, and P16. (**B**–**D**) Quantitative immunoblot analyses of Klotho, P21, and P16 expression. (**E**) Representative images of SA-β-gal staining. Scale bar = 50 μm. (**F**) Quantification of SA-β-gal staining. (**G**) Quantification of Lamin B1 staining. (**H**–**K**) qPCR analysis of SASP (*PAI1*, *TGF β*, *P53*, and *TNF α*) mRNA expression. (**L**) Representative images of Lamin B1 staining. The images contain the merge of Lamin B1 (red) and nuclei (blue). Scale bar = 20 μm. Data were expressed as means ± SEM for at least three independent experiments. *p* values were calculated using one-way ANOVA relative to the sham group. *** *p* < 0.001, ** *p* < 0.01, * *p* < 0.05 indicate the statistical significance compared to the sham group.

The results showed that the expression of cell-cycle-related proteins P16 and P21 gradually increased in the middle and late stages after kidney injury, with a significant difference between day 7 and day 14 (Figure 2A,C,D). In contrast, the expression of the anti-senescence protein Klotho gradually decreased and reached its lowest point on day 14 (Figure 2A,B). Compared with the sham group, the expression of senescence-associated β-galactosidase (SA-β-gal) was significantly increased at the middle stage of kidney injury (day 7), and was further increased at the later stage of kidney injury (day 14) (Figure 2E,F). The kidneys showed an overall increasing trend in the senescence-associated secretory phenotype (SASP) after UUO (Figure 2H–K), while the fluorescent expression of Lamin B1 gradually decreased. These results further confirmed that UUO could lead to renal cellular senescence (Figure 2G,L).

### 3.3. Increased Levels of Inflammation, Oxidative Stress, and Fibrosis

**Figure 3:**
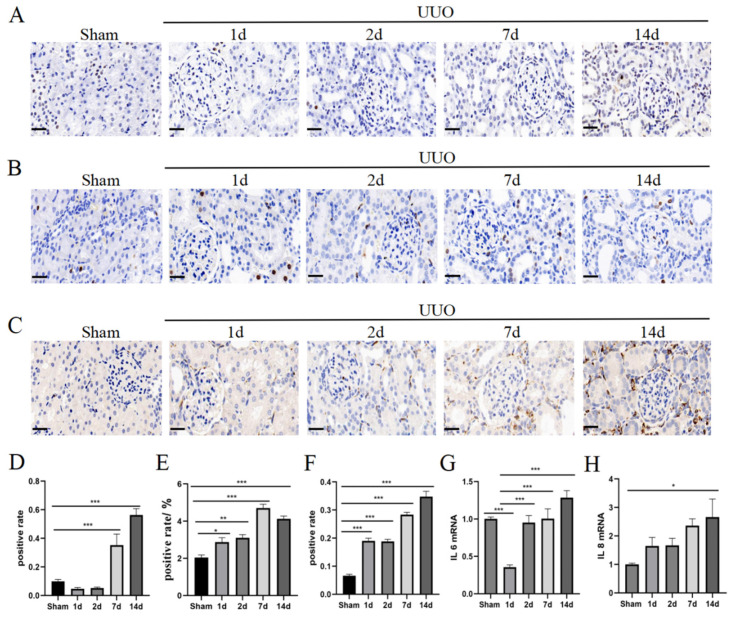
Increased levels of inflammation, oxidative stress, and fibrosis. (**A**) Representative images of Tunel staining. Positive results are brown in colour. Scale bar = 20 μm. (**B**) Representative images of Ki67 staining. Positive results are brown in colour. Scale bar = 20 μm. (**C**) Representative images of CD68 staining. Positive results are brown in colour. Scale bar = 20 μm. (**D**) Quantification of Tunel staining. (**E**) Quantification of Ki67 staining. (**F**) Quantification of CD68 staining. (**G**,**H**) qPCR analysis of IL6 and IL8 mRNA expression. Data were expressed as means ± SEM for at least three independent experiments. *p* values were calculated using one-way ANOVA relative to the sham group. *** *p* < 0.001, ** *p* < 0.01, * *p* < 0.05 indicate the statistical significance compared to the sham group.

**Figure 4:**
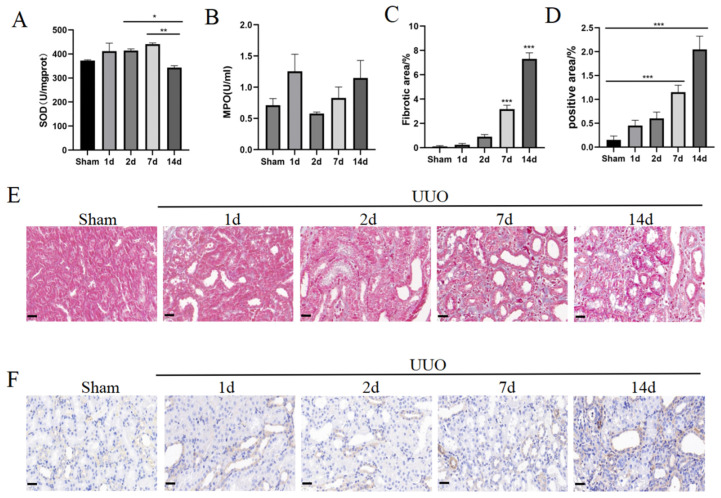
Increased oxidative stress and fibrosis in the kidney. (**A**) Quantification of SOD. (**B**) Quantification of MPO. (**C**) Quantification of Masson staining. (**D**) Quantification of α-SMA staining. (**E**) Representative images of Masson staining. Collagen fibres appear blue. Scale bar = 20 μm. (**F**) Representative images of α-SMA staining. Positive results are brown in colour. Scale bar = 20 μm. Data were expressed as means ± SEM for at least three independent experiments. *p* values were calculated using one-way ANOVA relative to the sham group. *** *p* < 0.001, ** *p* < 0.01, * *p* < 0.05 indicate the statistical significance compared to the sham group.

As the changes in renal functional indexes indicated the presence of compensatory repair, we further examined the apoptotic and proliferation capacity of the kidneys (Figure 3A,B,D,E). The results showed an increase in the overall level of apoptosis in renal tissue after UUO, along with a trend towards an increase in the level of proliferation. Although the level of proliferation slightly decreased at day 14, it was still higher than that at early stages of acute injury. This finding is consistent with changes in the renal functional indexes. In the early stages of acute injury, the kidneys exhibited a marked inflammatory response, which was further exacerbated by the prolongation of the duration of the obstruction at days 7 and 14 (Figure 3C,F–H). This inflammatory response may negatively affect the normal function and structure of the kidney. According to the results of the SOD activity assay, there was a significant decrease in SOD activity in the UUO group at the later stage of injury, indicating weakened antioxidant capacity (Figure 4A,B). On day 7, the kidney tissue began to show obvious collagen fiber deposition, fibroblast activation, and epithelial cell transformation, accelerating the fibrosis process of the kidney tissues (Figure 4C–F). By day 14, these changes became more pronounced, constituting clear evidence of a transition from acute injury to chronic fibrosis.

### 3.4. Elimination of Senescent Cells by ABT263 Treatment

**Figure 5:**
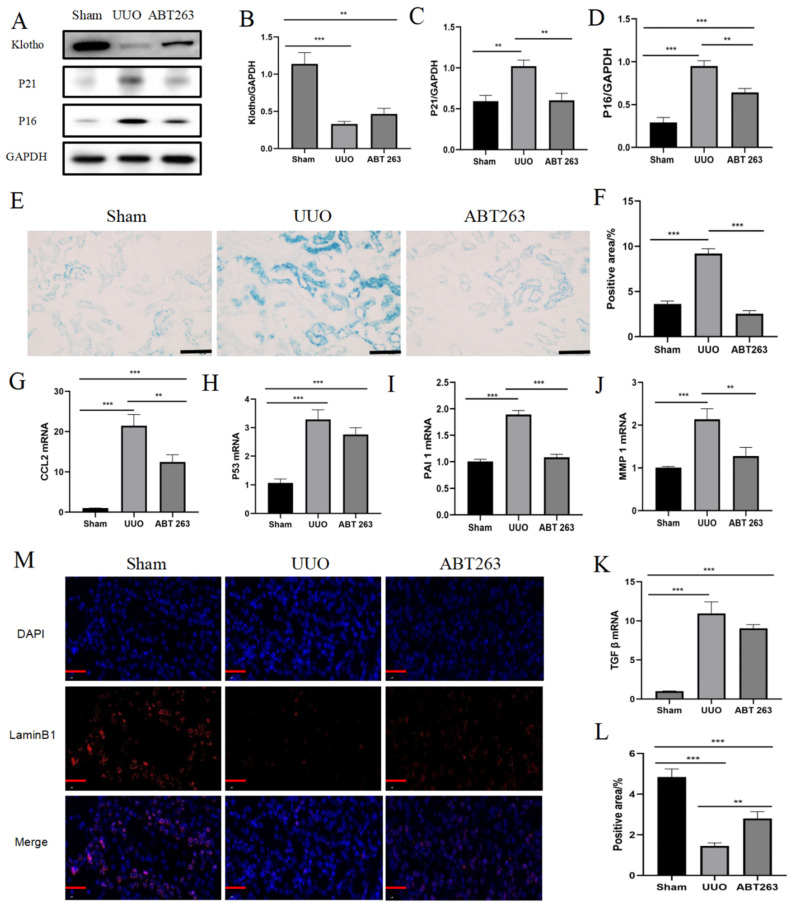
Elimination of senescent cells by ABT263 treatment. (**A**) Representative immunoblot images of Klotho, P21, and P16. (**B**–**D**) Quantitative immunoblot analyses of Klotho, P21, and P16 expression. (**E**) Representative images of SA-β-gal staining. Scale bar = 50 μm. (**F**) Quantification of SA-β-gal staining. (**G**–**K**) qPCR analysis of SASP (*CCL2*, *P53*, *PAI1*, *MMP1*, and *TGF β*) mRNA expression. (**L**) Quantification of Lamin B1 staining. (**M**) Representative images of Lamin B1 staining. Scale bar = 20 μm. The images contain the merge of Lamin B1 (red) and nuclei (blue). Data were expressed as means ± SEM for at least three independent experiments. *p* values were calculated using one-way ANOVA relative to the sham group. *** *p* < 0.001, ** *p* < 0.01 indicate the statistical significance compared to the sham group.

Under the treatment of the ABT263 drug, we observed that the decrease in senescence-associated protein Klotho, as well as the up-regulation of P16 and P21, were significantly suppressed (Figure 5A–D). In addition, ABT263 treatment led to a decrease in SA-β-gal positive regions, suggesting that ABT263 effectively eliminated the senescent cells (Figure 5E,F). Meanwhile, the reduced number of senescent cells led to a decrease in their secreted SASP content, including the expression of factors such as cytokines and extracellular matrix proteins (Figure 5G–K). In addition, the expression abundance of Lamin B1 was significantly increased, providing additional evidence for the intervention effect of ABT263 on renal cellular senescence (Figure 5L,M).

### 3.5. ABT263 Significantly Ameliorates the Progression of UUO-Induced Renal Disease

**Figure 6:**
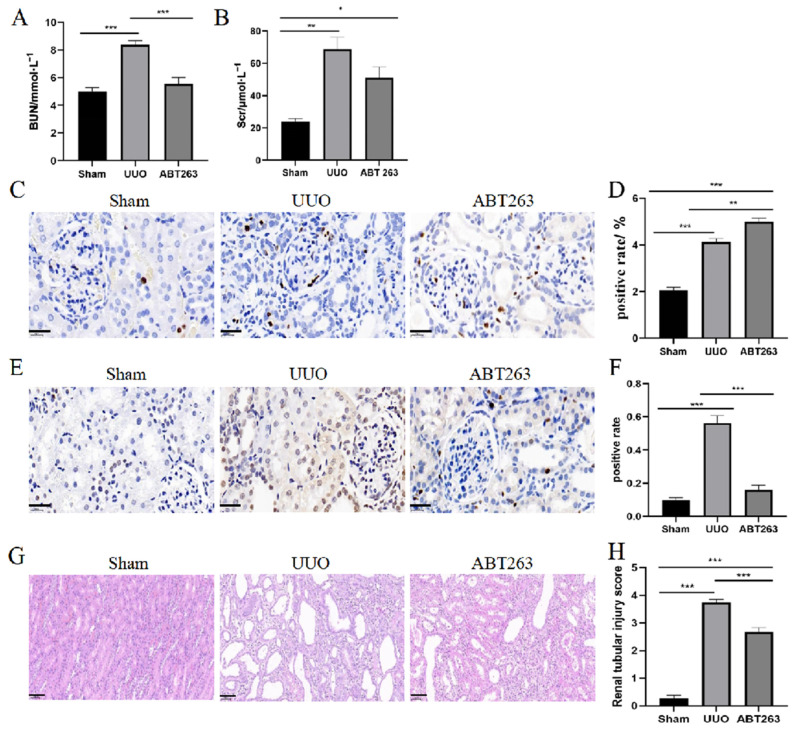
ABT263 significantly ameliorates the progression of UUO-induced renal disease. (**A**) Quantification of Bun. (**B**) Quantification of Scr. (**C**) Representative images of Ki67 staining. Positive results are brown in colour. Scale bar = 20 μm. (**D**) Quantification of Ki67 staining. (**E**) Representative images of Tunel staining. Positive results are brown in colour. Scale bar = 20 μm. (**F**) Quantification of Tunel staining. (**G**) HE staining. Scale bar = 50 μm. (**H**) Renal tubular injury score. Data were expressed as means ± SEM for at least three independent experiments. *p* values were calculated using one-way ANOVA relative to the sham group. *** *p* < 0.001, ** *p* < 0.01, * *p* < 0.05 indicate the statistical significance compared to the sham group.

**Figure 7:**
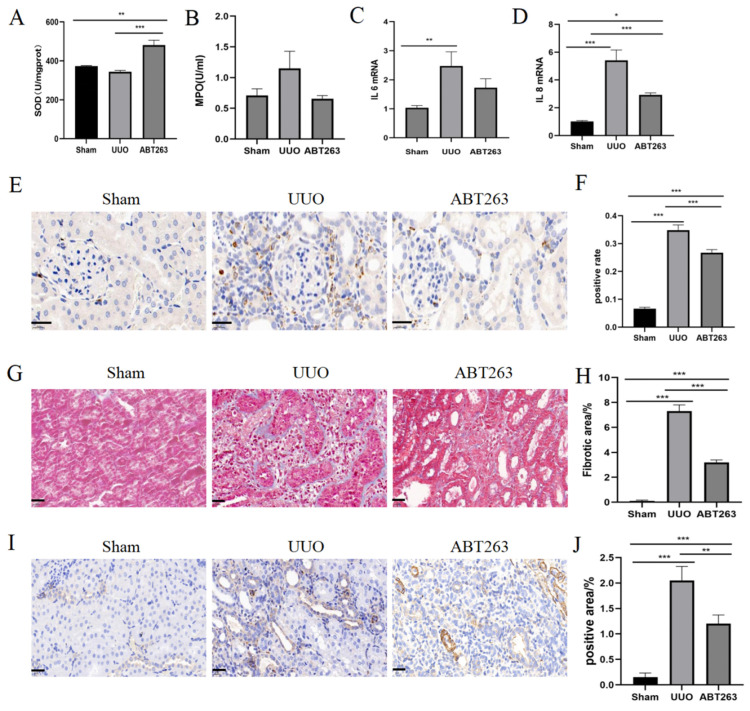
ABT263 significantly ameliorates UUO-induced inflammation and fibrosis in the kidney. (**A**) Quantification of SOD. (**B**) Quantification of MPO. (**C**,**D**) qPCR analysis of IL6 and IL8 mRNA expression. (**E**) Representative images of CD68 staining. Positive results are brown in colour. Scale bar = 20 μm. (**F**) Quantification of CD68 staining. (**G**) Representative images of Masson staining. Collagen fibres appear blue. Scale bar = 20 μm. (**H**) Quantification of Masson staining. (**I**) Representative images of α-SMA staining. Positive results are brown in colour. Scale bar = 20 μm. (**J**) Quantification of α-SMA staining. Data were expressed as means ± SEM for at least three independent experiments. *p* values were calculated using one-way ANOVA relative to the sham group. *** *p* < 0.001, ** *p* < 0.01, * *p* < 0.05 indicate the statistical significance compared to the sham group.

Renal function indicators, including creatinine and urea nitrogen concentrations, were significantly reduced after ABT263 drug intervention (Figure 6A,B). The overall apoptotic capacity of the kidney was significantly decreased, and the proliferation capacity was markedly improved (Figure 6C–F), which is conducive to the repair of kidney tissues after the injury (Figure 6G,H). ABT263 effectively eliminated the senescent cells, inhibited the secretion of SASP, suppressed oxidative stress and inflammatory cell infiltration, and enhanced the resistance of renal cells to oxidative stress (Figure 7A,B), thereby improving the cellular inflammatory microenvironment (Figure 7C–F). After the ABT263 intervention, the collagen fiber deposition in renal tissues was reduced and the overall fibrosis decreased (Figure 7G–J). These results suggested that the elimination of senescent cells by ABT263 intervention inhibited the oxidative stress and inflammation and prevented persistent damage in UUO-induced renal disease.

### 3.6. ABT263 Induces Apoptosis in Senescent Cells through the Caspase3 Pathway

**Figure 8:**
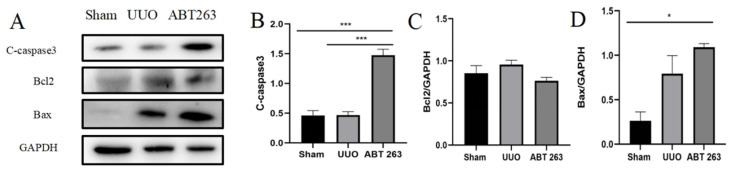
ABT263 induces apoptosis in senescent cells via the Caspase3 pathway. (**A**) Representative immunoblot images of C-caspase3, Bcl2, and Bax. (**B**–**D**) Quantitative immunoblot analyses of C-caspase3, Bcl2, and Bax. Data were expressed as means ± SEM for at least three independent experiments. *p* values were calculated using one-way ANOVA relative to the sham group. *** *p* < 0.001, * *p* < 0.05 indicate the statistical significance compared to the sham group.

ABT263 is a class of Bcl2 inhibitors, which are proteins associated with apoptosis. In order to confirm whether the elimination of senescent cells by ABT263 is related to the pro-apoptotic signaling pathway, a Western blot was used to detect the apoptosis-related protein C-caspase3. The results showed a significant increase in the expression of the C-caspase3 protein in the ABT263 group. In addition, a decreasing trend was observed in the expression of the anti-apoptotic protein Bcl2, while the expression of the pro-apoptotic protein Bax increased significantly. These results confirmed that ABT263 selectively eliminated senescent cells by inducing apoptosis (Figure 8A–D).

## 4. Discussion

This study observed a series of senescence-associated phenotypic changes in the injured kidney during the progression from UUO-induced acute injury to chronic fibrosis, suggesting the emergence of cellular senescence. The results showed that cellular senescence disrupted the function of the kidney, affecting its overall apoptotic and proliferative capacity, and that the levels of inflammation and oxidative stress were increased, leading to renal fibrosis. Targeted anti-senescence drug ABT263’s intervention induced the apoptosis of senescent cells through the caspase3 pathway, effectively reducing the number of senescent cells, enhancing the proliferation capacity of the kidney, alleviating renal inflammation and oxidative stress, reducing the degree of fibrosis, and contributing to the tissue and functional repair of the kidney after injury. This further demonstrates that the targeting of cellular senescence has the therapeutic potential to protect the kidney from UUO-induced injury.

When an acute injury occurs in the kidney, timely intervention and treatment can effectively restore the renal function and allow patients to recover gradually. If the acute injury is inadequately managed or left untreated, it may lead to chronic renal fibrosis. Hydronephrosis induced by UUO is a common cause of kidney injury [21]. When UUO occurs, urine cannot flow out normally, resulting in increased pressure within the kidney and the inhibition of cellular metabolic activity and function [22]. In such a case, there is a progression from acute renal decompensation to a gradual protection of its function through contralateral renal compensation, ultimately leading to contralateral renal decompensation as well [23]. As with previous studies, our study identified cellular senescence in a model of UUO-induced kidney injury [15,24]. With the extension of obstruction, the level of senescence increases, the tissue structure and function of the kidney are destroyed, inflammation and oxidative stress levels increase, and chronic fibrosis appears. Compared with previous studies, this study systematically selected different time points for experiments after UUO to explore the role of cellular senescence during the transition from acute injury to chronic fibrosis, a period which is often neglected.

Cellular senescence is one of the important factors in the progression of renal injury, and aggravates renal injury through mechanisms such as inflammatory response and oxidative stress, ultimately leading to chronic fibrosis [25,26]. The inflammatory response is a key factor in the process of cellular senescence [27,28]. Specifically, senescent cells can induce inflammation by secreting SASPs such as pro-inflammatory cytokines, chemokines and proteases, altering the cellular microenvironment and inducing senescence in adjacent cells [29,30,31]. Cytokines in SASPs, such as *IL6*, *IL1β*, and *TNF α*, can activate the NF-κB signaling pathway and other inflammatory pathways, exacerbating inflammatory damage in renal tissues [32,33]. Even worse, inflammatory mediators and cytokines will promote the proliferation of fibroblasts and collagen synthesis, advancing the development of renal fibrosis and ultimately leading to impaired renal function [31,34,35]. At the same time, cellular senescence can also lead to disturbances in intracellular redox balance and the accumulation of oxidized substances, exacerbating renal structural and functional impairments while expediting the fibrotic process [35,36]. These processes interact with each other, and the kidney initiates a defense response to various injuries. The maladaptive repair process promotes the phenotypic transformation of renal cells, the proliferation of renal fibroblasts, and abnormal extracellular matrix deposition, forming a vicious circle that continuously aggravates renal injury and ultimately leads to chronic fibrosis. In this study, the proliferative capacity of cells usually tended to increase first and then decrease. This is because the initial injury can trigger an inflammatory response and oxidative stress, stimulating kidney cell proliferation. With the intensification of inflammatory response and oxidative stress, the proliferative capacity of kidney cells gradually decreases. Meanwhile, the persistent inflammatory response and oxidative stress will further aggravate the degree of kidney fibrosis. Therefore, in the later stages of the injury, cell proliferation can also be restricted, leading to reduced cell numbers and the destruction of the tissue structure in the kidney, further aggravating the damage [37]. Therefore, a comprehensive understanding of the role of cellular senescence in chronic fibrosis can help reveal the mechanism of renal disease development, offering novel insights and strategies for treatment and prevention.

ABT263 is a common class of Bcl2 inhibitors, and it is also a common class of senolytic drugs [38,39]. The treatment of anti-senescence drug ABT263 in this study has effectively eliminated senescent cells through the caspase3 apoptosis signaling pathway, alleviated inflammation and oxidative stress in UUO kidneys, inhibited fibroblast activation in UUO kidneys, and reduced the degree of renal fibrosis [38,39]. Studies have also proved that ABT263 is a selective anti-senescence drug, and senescent cells are more sensitive to ABT263 treatment than non-senescent cells [38]. After kidney injury, not all cells undergo severe senescence. Among the affected cell types, renal tubular epithelial cells suffered the most severe injuries, yet the altered microenvironment of the kidney following drug intervention rescued most cells from apoptosis [40]. Thus, although ABT263 activated the caspase3 apoptotic pathway, apoptosis in the kidney as a whole was still able to decrease after drug intervention. These findings demonstrated that the intermittent use of ABT263 to eliminate senescent cells can effectively ameliorate the transition from early acute injury to chronic fibrosis induced by UUO, providing a new direction and strategy for the clinical treatment of UUO-induced renal disease.

There are some limitations of this study that should be addressed in future studies. This study did provide valuable insights into the study of renal disease, but it was conducted on animals, and caution must be exercised when applying the findings to humans. This is due to human physiology and disease progression not being exactly the same as in animals; it is insufficient to represent humans by solely depending on animal experiments. Moreover, although ABT263 has been proven to eliminate senescent cells in animal models, the specific uses of ABT263 treatment, including drug metabolism, optimal dose, and potential adverse effects in clinical trials, are still unclear and require further research. These factors may have a significant impact on treatment efficacy and patient safety, therefore requiring much more caution. In addition, this study looks at the transition phase between acute injury and chronic fibrosis, which lasts only 2 weeks in the ABT263 treatment, and the long-term application effect of ABT263 will be further explored in future studies. This study did not consider the bilateral effect of senescence on the organism, namely that acute senescence has a repairing effect on the organism. Therefore, early intervention and control of the development of cellular senescence during treatment may help to slow the progression of chronic fibrosis and protect renal function.

In conclusion, this study demonstrates that UUO-induced kidney injury can be effectively attenuated by eliminating senescent cells using the anti-senescence drug ABT263, highlighting the critical role of cellular senescence in the transition from acute injury to chronic fibrosis. This study also reveals the potential of targeted drug therapy for renal diseases, which provides a theoretical basis for new targets in clinical intervention in the treatment of renal diseases and offers new research perspectives for the repair of renal injury.

## Figures and Tables

**Table 1:** Primer sequence.

Gene	Forward Primer	Reverse Primer
*GAPDH*	AGGTCGGTGTGAACGGATTTG	TGTAGACCATGTAGTTGAGGTCA
*IL6*	ATTACCAAACTCAGCTAAACGGG	ACCAGGCGAGGGATCTCAG
*IL8*	TGACCATGAGACACTGTGGC	GAAGAGCACGGGTCCTTTGA
*IL10*	AAGGGTTACTTGGGTTGCCA	AAATCGATGACAGCGTCGCA
*P53*	ATGGAGGATTCACAGTCGGATAT	CGCTGTGGTGGGCAGAATAT
*MMP1*	GGAAAGGCTTTTCGACTTGCT	GGGTTCCATTGATGGTCCAGAA
*TGF β*	GCTGAACCAAGGAGACGGAATA	GCAGGTGTTGAGCCCTTTCC
*TNF α*	CCACCACGCTCTTCTGTCTACTG	TGGGCTACGGGCTTGTCACT
*PAI1*	AGTGGTGACAAACGGCTACTA	ACCGGAGGGCATACAGTTCTT
*CCL2*	TCTCAGCCAGATGCAGTTAATG	ACTTCTGGACCCATTCCTTATTG

## Data Availability

The data used in this study is available from the corresponding author upon request.
